# Enzymatic Cellulose Hydrolysis: Enzyme Reusability and Visualization of β-Glucosidase Immobilized in Calcium Alginate

**DOI:** 10.3390/molecules191219390

**Published:** 2014-11-25

**Authors:** Chien-Tai Tsai, Anne S. Meyer

**Affiliations:** Center for Bioprocess Engineering, Department of Chemical and Biochemical Engineering, Technical University of Denmark, DK-2800 Kgs. Lyngby, Denmark; E-Mail: aaron0115@gmail.com

**Keywords:** immobilization, β-glucosidase, alginate, lignocellulose, confocal microscopy

## Abstract

The high cellulase enzyme dosages required for hydrolysis of cellulose is a major cost challenge in lignocellulosic ethanol production. One method to decrease the enzyme dosage and increase biocatalytic productivity is to re-use β-glucosidase (BG) via immobilization. In the present research, glutaraldehyde cross-linked BG was entrapped in calcium alginate gel particles. More than 60% of the enzyme activity could be recovered under optimized conditions, and glutaraldehyde cross-linking decreased leakage of BG from the calcium alginate particles. The immobilized BG aggregates were visualized by confocal laser scanning microscopy (CLSM). The CLSM images, which we believe are the first to be published, corroborate that more BG aggregates were entrapped in the matrix when the enzymes were cross-linked by glutaraldehyde as opposed to when they are not cross-linked. The particles with the immobilized BG were recycled for cellulase catalyzed hydrolysis of Avicel. No significant loss in BG activity was observed for up to 20 rounds of reaction recycle steps of the BG particles of 48 h each, verifying a significant stabilization of the BG by immobilization. Similar high glucose yields were obtained by one round of enzymatic hydrolysis of hydrothermally pretreated barley straw during a 72 h reaction with immobilized BG and free BG.

## 1. Introduction

In order to produce bioethanol from lignocellulosic biomass, the 1,4-β-d-glycosidic linkages in cellulose must be cleaved to release glucose for the subsequent fermentation. Cellulases (and hemicellulases) produced by the fungus *Trichoderma reesei* (an anamorph of *Hypocrea jecorina*) (Rut C-30) are currently the main cellulases used in the industrial development of second generation bioethanol [1]. *T. reesei* secretes three main types of cellulose-degrading enzymes for catalyzing this enzymatic hydrolysis process: (1) endo-1,4-β-d-glucanase (EG, EC 3.2.1.4), which catalyzes random cleavage the internal β-1,4 bonds in the cellulosic polymers; (2) exo-1,4-β-d-glucanase or cellobiohydrolase (CBH, EC 3.2.1.91), which catalyzes hydrolysis of the second outermost β-1,4 bonds by attacking the cellulose from the ends only, releasing mainly cellobiose; CBH1 (or Cel7A) attacks the reducing ends, whereas CBHII (or Cel6A) attacks the non-reducing ends of the cellulose polymers, and (3) β-glucosidase (BG, EC 3.2.1.21) which catalyzes the hydrolysis of cellobiose and other short cellulo-oligomers to liberate glucose [2]. That *T. reesei* produces these three classic categories of cellulases in addition to a large set of other glycoside hydrolases (e.g., several hemicellulases, chitinases) and some expansin type proteins have now been corroborated by gene and functionality analyses [3,4]. For the sake of completion, it should be mentioned also, that a new type of lytic polysaccharide monooxygenases (EC 1.-.-.-), now categorized as auxiliary family activity 9, AA9, which catalyze oxidative cleavage of 1,4-β-linkages in cellulose are known [5]; the endo-β-1,4-glucanase IV (“Cel61A”) of *T. reesei* Rut C-30 has now been categorized in family AA9 [6]. Nevertheless, the original classification of three main groups of hydrolytic cellulases produced by *T. reesei* remains [4].

Cellobiose in particular, and to a certain extent also glucose, exerts significant product inhibition on the enzymatic cellulose hydrolysis accomplished by these enzymes [7]. Improvements in the catalytic efficiencies of EG and CBH can thus be seen following removal of the strongly inhibitory cellobiose from the reaction [7,8]. Whereas *T. reesei* does express BG activity, the activity is not sufficiently high in *T. reesei-*derived enzyme preparations to catalyze hydrolysis of the accumulated cellobiose because a major part of the BG activity is bound to the fungal mycelium and hence not recovered during industrial cellulase production [9]. Additional BG is therefore required to achieve sufficiently high glucose yields as well as for securing satisfactory efficiencies of EG and CBH. Overall, higher glucose yields may be attained through increased dosing of EG, CBH and BG, but addition of higher enzyme dosages of course results in higher enzyme costs, and the high cost of enzymes is already a significant barrier to economical production of ethanol from lignocellulosic biomass [10,11]. In addition to producing enzymes economically and improving the *T. reesei* cellulase enzyme expression, the cellulase productivity can be improved by designing better enzyme mixtures [1,9,12] or by developing novel engineered fungal strains to produce more efficient lignocellulolytic enzyme systems [13]. However, recycling or re-using of the enzymes can be used to reduce the total enzyme loading [14,15]. Recent work has proven that re-use of cellulases by recycling the insoluble solids fraction can increase enzyme productivity; However, the recycling of the insoluble solids is accompanied by process challenges since the total reaction volumes, solid concentrations, and the lignin content in the insoluble residues will also keep increasing during this type of processing [15]. This is why enzyme immobilization for enzyme re-use represents an attractive option, because enzyme performance, such as enzyme activity, selectivity, and stability may also improve via the immobilization [16,17,18].

Immobilization of EGs and CBHs will invariably cause the enzyme catalysis to be mass transfer limited due to the slow diffusion of the insoluble substrate making this option futile. In contrast, the water soluble properties of cellobiose and the much faster mass transfer of this substrate and its hydrolysis products (glucose) makes BG a much better candidate for immobilization.

Different BG immobilization technologies and support materials have already been investigated including, e.g., calcium alginate [19,20,21], polyacrylamide [22], soil humates [23], and silica [24]. Among those, calcium alginate entrapment [19,20,21] is a simple and inexpensive method for enzyme immobilization. This technology furthermore allows easy enzyme recycling by simple recovery of the calcium alginate beads (containing the immobilized enzyme) from the reaction slurry, e.g., by arranging that the beads are physically confined in the reaction mixture to allow repeated or continuous use. However, leakage of enzyme from the calcium alginate matrix has been reported [19,25,26]. To overcome this problem, a strategy involving pre-immobilization by cross-linking prior to further immobilization in calcium alginate beads may be used. Glutaraldehyde is a well-known agent for intra- and intermolecular cross-linking of proteins and enzymes [27,28,29]. Glutaraldehyde can also cross-link BG to form larger enzyme aggregates [19,30]. In relation to cross-linking it has been proposed that addition of bovine serum albumin (BSA) may reduce enzyme inactivation during the cross-linking reaction [31].

Most of the available research concerning BG immobilization for cellulose hydrolysis has focused on fundamental analyses of enzymatic parameters. There is limited knowledge of extensive recycling of immobilized BG (IMBG) through prolonged hydrolysis cycles of cellulose-catalyzed degradation of cellulose or lignocellulose. Moreover, the distribution of the entrapped BG in the calcium alginate polymer network of the immobilization material has to our knowledge not been examined either. The objective of the present work was thus to evaluate the extended recyclability of BG immobilized in calcium alginate particles with the purpose of assessing the options for improving enzyme productivity during enzymatic cellulose hydrolysis. A sub-purpose was to examine the significance of the cross-linking with glutaraldehyde for the immobilization efficiency, notably with respect to avoiding leakage of enzyme activity from the calcium alginate beads. Hence, in this research, BG was cross-linked with glutaraldehyde to yield cross-linked enzyme aggregates which were then entrapped in 3.75% calcium alginate. The residual activity and recyclability of the immobilized BG were investigated. Enzyme aggregation and distribution within the alginate matrix were visualized by confocal laser scanning microscopy (CLSM).

## 2. Results and Discussion

### 2.1. Effects of Immobilization Conditions on Residual Activity

**Figure 1:**
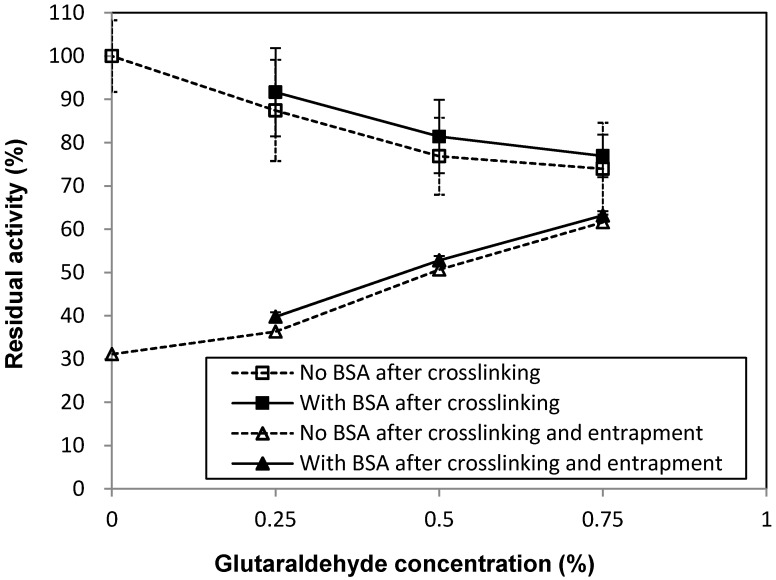
Residual activity of BG after glutaraldehyde cross-linking (3 replications) and following the full enzyme immobilization process (crosslinking + entrapment), respectively. Codes A–G refer to cross-linking conditions as given in Table 1.

Prior to calcium alginate entrapment, the BG was cross-linked using different glutaraldehyde concentration conditions, with or without BSA (Table 1). The purpose of cross-linking was to aggregate the BG into larger particles, thereby hindering their diffusion from the calcium alginate matrix. In practice, glutaraldehyde may react with the enzyme during polymerization in such a way that enzymatic activity is reduced. Overall, the use of increased levels of glutaraldehyde during the cross-linking step did result in gradually greater inactivation of BG immediately after cross-linking (Figure 1).

**Table 1:** Cross-linking conditions for β-glucosidase prior to enzyme immobilization in calcium alginate beads.

	BG (mg/mL)	BSA (mg/mL)	Glutaraldehyde (%)
A	7.33	0	0.75
B			0.5
C			0.25
D			0
E		3	0.75
F			0.5
G			0.25

Addition of BSA with glutaraldehyde (Table 1), based on the hypothesis that BSA might provide some amine groups for cross-linking, thus reducing the probability that BG active sites would be blocked by glutaraldehyde, did not affect the residual activity (Figure 1).

After cross-linking, the cross-linked BG enzymes were entrapped in calcium alginate (3.75% by weight of calcium alginate). In most previous literature, the concentration of calcium alginate employed has typically been between 2%–3%. However, in order to reduce leakage of the enzyme and obtain appropriate mechanical strength of the beads, a concentration of 3.75% was used. At this concentration, the viscosity of the system was high and the resulting beads were globular/slightly droplet formed and had a diameter of 4–5 mm. Although increasing the concentration of glutaraldehyde gradually inactivated the BG, less enzyme activity leaked from the beads with higher glutaraldehyde levels (Figure 1), an effect most likely due to improved entrapment of the enzyme aggregates by the calcium alginate matrix. The optimal conditions, which resulted in more than 60% residual activity, were obtained when the enzyme was cross-linked with 0.75% glutaraldehyde and then entrapped in 3.75% calcium alginate, regardless of BSA addition (Figure 1, sample A (or E)).

### 2.2. K_m_ and V_max_ of BG

**Figure 2:**
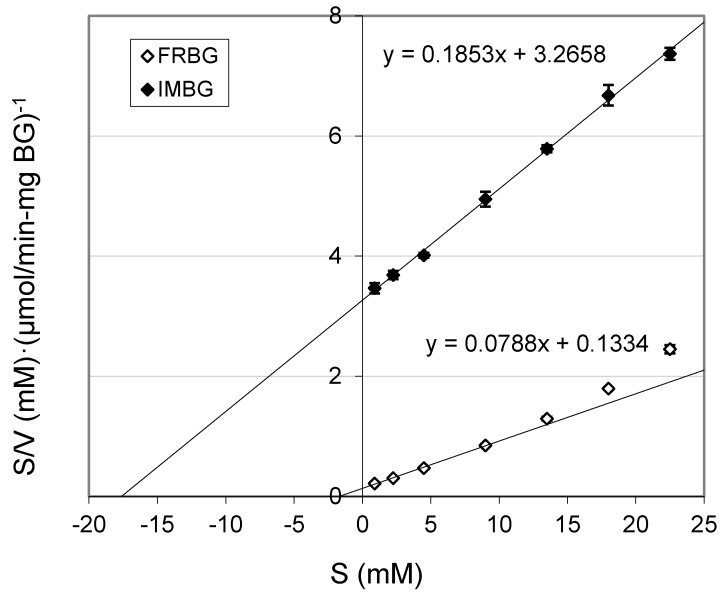
A Hanes-Woolf plot of free (FRBG) and immobilized BG (IMBG). The straight line for the FRBG was fitted and extrapolated by linear regression from the substrate concentrations of cellobiose between 0 and 9 mM only.

The K_m_ of FRBG and IMBG were determined to be 1.7 and 17.6 mM, respectively (Table 2) from linear regression of different substrate levels of cellobiose in Hanes-Woolf plots (Figure 2).

**Table 2:** K_m_ and V_max_ of β-glucosidase on cellobiose: Free (FRBG) and immobilized (IMBG).

	K_m_ (mM)	V_max_ (μmol/(min·mg BG))
FRBG	1.70 ± 0.12	12.71 ± 0.26
IMBG	17.62 ± 0.11	5.39 ± 0.06

Hence, the K_m_ of the IMBG was significantly larger than that of the free enzyme, and the V_max_ of the FRBG, at 12.7 μmol/(min·mg BG), was about 2.4 times higher than that of the IMBG (Table 2). Both the larger K_m_ and the lower V_max_ of the immobilized enzyme are in complete accord with the expected diffusion limitations induced by the porous immobilization matrix. The markedly elevated K_m_ is presumably also due to the likely product inhibition by glucose, which will enhance the K_m_ by a factor of (1 + [P]/K_i_), producing an apparent K_m_, K_m,app_, equal to K_m,app_ = K_m_·(1 + [P]/K_i_), where [P] indicates the glucose product concentration and K_i_ is the inhibitor dissociation constant for the enzyme:product complex consistent with competitive Michaelis-Menten product inhibition kinetics [7]; this interpretation agrees well with previous literature data for immobilized β-glucosidase [19].

### 2.3. Repeated Hydrolysis and Stability of the Recycled, Immobilized Enzyme

**Figure 3:**
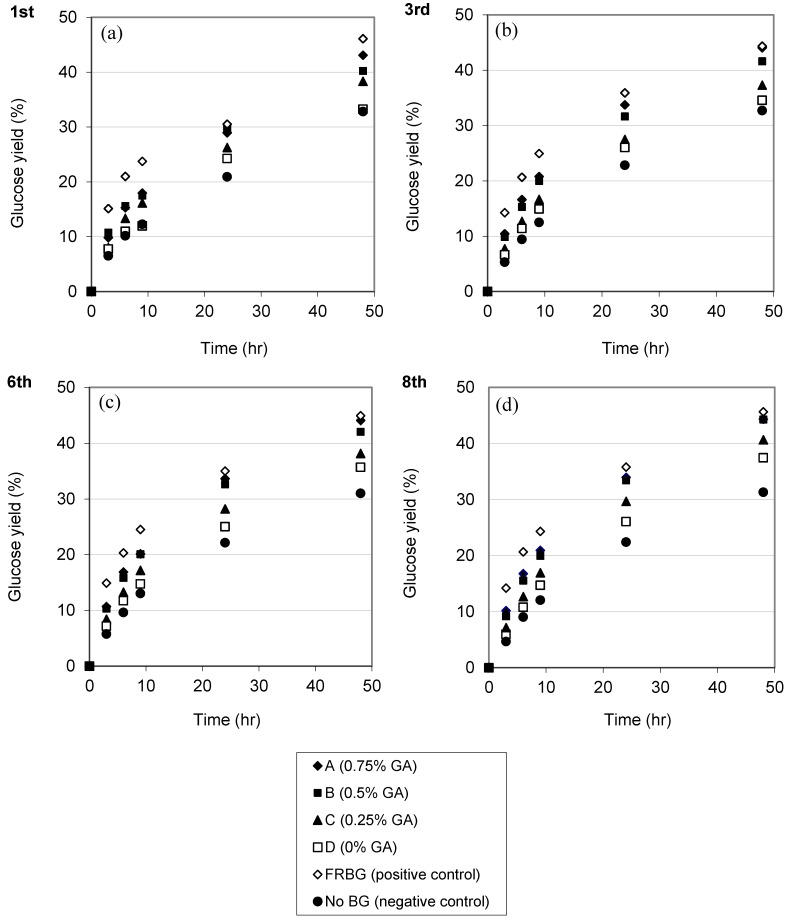
Time course kinetics of free and immobilized BG during repeated hydrolysis reaction. (**a**), (**b**), (**c**) and (**d**) are 1st, 3rd, 6th and 8th rounds respectively. A, B, C and D are IMBG prepared by different cross-linking conditions (Table 1) (Data for cross-linking with BSA not shown). FRBG and No BG are positive and negative controls respectively.

In order to evaluate the reusability and catalytic enzyme stability under hydrolytic reaction conditions, the IMBG was recycled repeatedly following hydrolysis using Celluclast 1.5 L catalysis for 48 h on 10% w/w Avicel at 50 °C, pH 4.8 during shaking. Figure 3 compares time course curves depicting the hydrolysis kinetics of IMBG, with BG cross-linked with different concentrations of glutaraldehyde, as well as *versus* the FRBG (positive control, 13 CBU/g-substrate) and a sample without BG (negative control). Some glucose was released from the negative control, which is likely a result of the Celluclast 1.5 L preparation possessing some BG activity (consistent with the preparation harbouring 10 CBU/mL) and agrees with results reported by others as well [26]. Each hydrolysis reaction thus contained ~2.5 CBU/g-substrate due to Celluclast 1.5 L, calculated as follows:

Enzyme activity of Celluclast 1.5 L is 65 FPU/mL and 10 CBU/mL respectively, and the dosage in the reaction was 16 FPU/g-substrate. The volume of Celluclast 1.5 L added was:

16 (FPU/g-substrate)/65 (FPU/mL) = 0.246 (mL/g-substrate)

The dosage of CBU in the reaction contributed from Celluclast 1.5 L therefore was:

0.246 (mL/g-substrate) × 10 (CBU/mL) = 2.46 CBU/g-substrate

**Figure 4:**
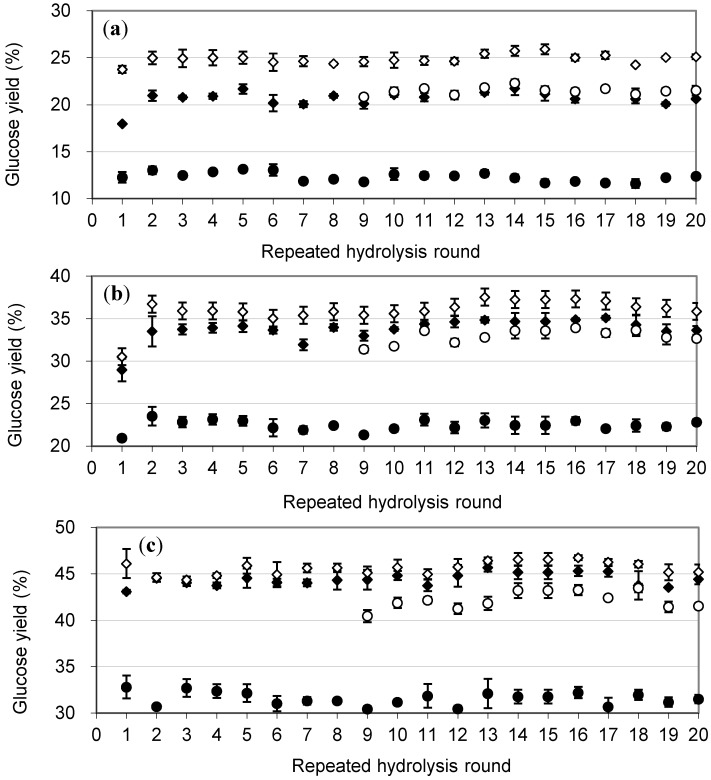
Glucose yields obtained with addition of the immobilized, glutaraldehyde cross-linked BG in calcium alginate beads on Celluclast 1.5 L catalyzed degradation of Avicel after repeated rounds of extended hydrolysis *vs.* addition of free BG. (**a**), (**b**) and (**c**) are glucose yields at 9, 24 and 48 h. “A” is the IMBG prepared under conditions as in Table 1. ♦ A; ◊ Free BG; ○ ½ Free BG; ● No BG. Data points are shown as averages ± s.d.

The quantity of IMBG employed in each reaction corresponded to 2.25 g of immobilized beads. In the event that no BG was inactivated during cross-linking or lost due to leakage after entrapment, the dosage should have corresponded to 13 CBU/g-substrate. However, some activity was lost during the immobilization process. As such, the active IMBG remaining in each system was less than 13 CBU/g-substrate (e.g., the dosage of active IMBG in sample A was around 13 × 0.62 = 8 CBU/g-substrate). Prior to use in hydrolysis reactions, the beads were incubated in buffer for 8 days and changed buffer three times to remove non-entrapped enzymes, including mainly enzyme molecules not cross-linked by glutaraldehyde to diffuse out of the beads. This means that the glucose released during the reaction (Figure 3 and Figure 4) was a hydrolysis product of the entrapped, immobilized BG. The use of increasing glutaraldehyde concentrations during cross-linking resulted in increased reaction efficiencies within the range investigated. This finding also corroborates the results showing that more BG was retained in the matrix when cross-linking efficiency was higher (Figure 1). It should be noted that some BG apparently remained entrapped within the beads even without glutaraldehyde cross-linking treatment, since more glucose was released from the non-glutaraldehyde treated BG samples (Figure 3, sample D) than from the negative control.

All IMBG samples prepared using different levels of glutaraldehyde were very stable when recycled up to 8 times, suggesting that the BG activity was stable as long as the BG aggregates were entrapped in the matrix. It has been reported previously that entrapped uncross-linked BG had a higher thermal stability than the free enzyme [32]. It is also known that glutaraldehyde treated BG has higher thermostability than the corresponding free BG [29,33]. These examples indicate that the IMBG was likely stabilized by molecular interactions, which is the most widely accepted mechanism for stabilization for glutaraldehyde cross-linked immobilized enzymes.

The glucose yields achieved following each round of re-use with addition of the IMBG prepared through cross-linking with 0.75% glutaraldehyde (*i.e*., sample A, Table 1) after 20 rounds of recycling are shown in Figure 4. Comparison of these three time points allows for a more accurate assessment of enzyme performance. In most enzymatic hydrolysis reactions, the reaction rates slow down significantly after 12 h due to the depletion of substrate and accumulation of product. As 48 h corresponds to most industrial processing times for lignocellulose hydrolysis in bioethanol processing (48–72 h), the hydrolytic efficiency at this late stage reflect the overall performance. Glucose yields from the beginning and the middle stages of the reaction reveal the initial reaction rate and the rate decrease as the reaction proceeds. The glucose yields (Figure 4) were recorded after completion of each repeated reaction round. There was no significant decrease in efficiency until the 20th round after which the experiment was ceased (Figure 4). To evaluate the performance of IMBG during the extended repetitions, *i.e.*, from the 9th round, a hydrolysis reaction containing only 6.5 CBU/g-substrate of FRBG was used as an additional “1/2 positive control”, *i.e.*, a base line in the case half of “positive control” FRBG were inactivated. After 9 h, the reaction rate of the 1/2 positive control appeared faster than the IMBG due to internal diffusion limitations (Figure 4). In contrast, glucose yields of the IMBG were higher after 24 to 48 h. This means that the apparent dosage of IMBG was still higher than 6.5 CBU/g-substrate even during the extended rounds of recycling, confirming the high stability of the IMBG.

### 2.4. Enzymatic Hydrolysis of Hydrothermally Pretreated Barley Straw

**Figure 5:**
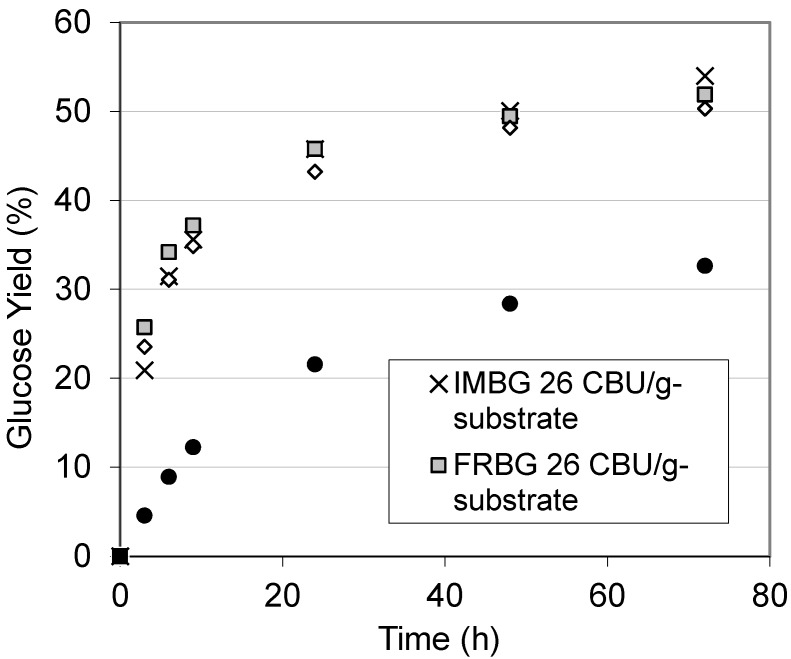
Time course of enzymatic hydrolysis of hydrothermally pretreated barley straw (5% dry matter w/v, reacted at 50 °C, pH 4.8) using a base dose of 8 FPU/g-substrate Celluclast 1.5 L with free (FRBG) or immobilized BG (IMBG) added at different dosages (13 or 26 CBU/g-substrate). Data points are shown as averages ± s.d.

To evaluate the performance of the IMBG on genuine lignocellulose, the IMBG was also evaluated for hydrolysis of pretreated barley straw (Figure 5).

According to compositional analysis, the potential glucose content in the pre-treated barley straw was 66% (w/w). The dosage of Celluclast 1.5 L employed in this hydrolysis reaction was 8 FPU/g-substrate (this dosage had been determined as appropriate in preliminary experiments to compare to the 16 FPU/g-substrate with Avicel as substrate). The initial reaction rate using FRBG was faster than that achieved with the IMBG, and can be explained by the significant difference in the K_m_ values resulting from the diffusional limitations of the IMBG matrix. However, the final yields of glucose obtained from the FRBG and IMBG reaction systems after extended hydrolysis time were similar (24 h, 48 h, 72 h, Figure 5). This proves that the IMBG can act efficiently as well on a genuine lignocellulosic substrate.

**Figure 6:**
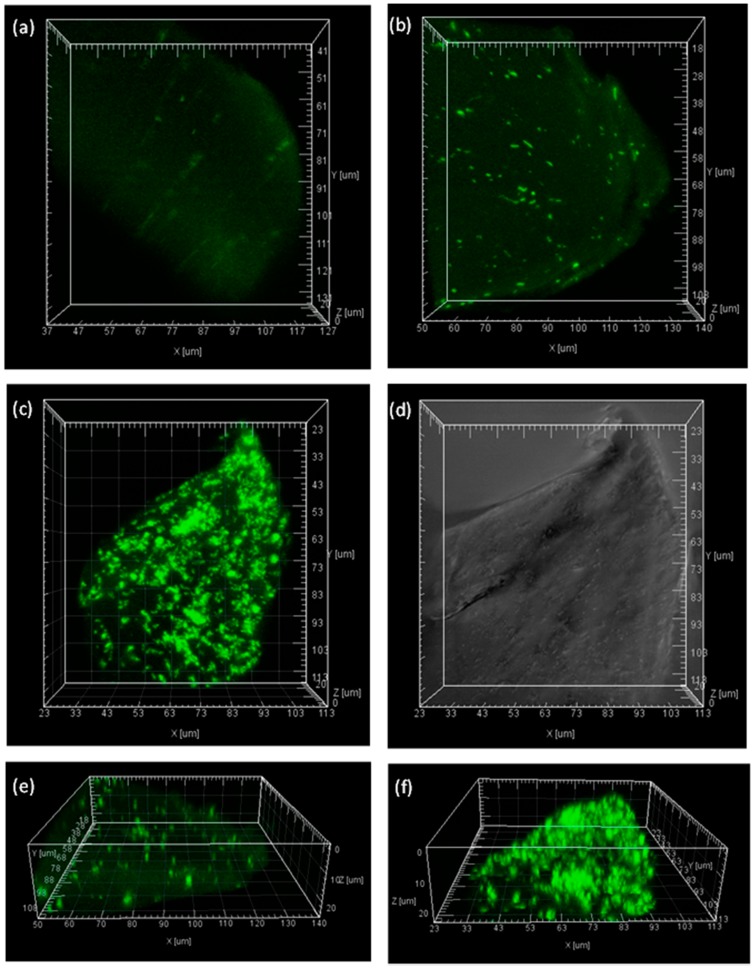
Confocal Laser Scanning Microscopy images of BG distribution inside calcium alginate beads visualized using fluorescein. (**a**) Calcium alginate only; (**b**) and (**e**) IMBG without glutaraldehyde treatment; (**c**) and (**f**) IMBG after 0.75% glutaraldehyde treatment; (**d**) Image of (**c**) but without fluorescence.

### 2.5. Visualization of the Immobilized BG inside Calcium Alginate Particles

In order to visualize the distribution of the enzymes inside the calcium alginate matrix, different IMBG samples were stained with fluorescein and observed under CLSM. The CLSM of fluorescein stained BG enzymes, which had not been treated with glutaraldehyde, but were inside calcium alginate beads, appeared as distinct dots that were relatively evenly distributed inside the IMBG matrix (Figures 6b,e). The faint background stain (Figure 6a) presumably stems from weak binding of the fluorescein to the calcium alginate. The IMBG treated with 0.75% glutaraldehyde showed a very strong signal, which we ascribe as being due to the presence of aggregates (Figures 6c,f), with most aggregates measuring >1 µm. This visual is consistent with the finding that BG leakage decreased due to the entrapment of cross-linked aggregates within the matrix. The data of the glutaraldehyde treated IMBG thus reveal that the IMBG enzymes were evenly distributed inside the calcium alginate beads (Figure 6c). The confirmation of the relatively even distribution of the immobilized, glutaraldehyde cross-linked BG aggregates inside the calcium alginate matrix is significant and corroborates the classic textbook comprehension of how immobilized enzymes are distributed inside porous particles and in turn how the kinetics may be affected by the internal diffusion [34]. Hence, this visualization also supports the kinetics observations in relation to the K_m_ and the V_max_ of the IMBG, namely that the reaction is essentially under intrinsic diffusion control since both the product diffusion rate and the substrate diffusion rate inside the beads are most likely decreased. However, because of the long reaction time of each round of hydrolysis of cellulose hydrolysis, this potential kinetic restriction of the IMBG catalyzed hydrolysis did affect the outcome of the hydrolysis reaction in practice, and the immobilized BG performed on par with the free enzyme during each round of hydrolysis (Figure 4).

## 3. Experimental Section

### 3.1. Chemicals and Enzymes

Sodium alginate, glutaraldehyde, Avicel PH-101 cellulose, sodium acetate, sodium azide, adenosine 5'-triphosphate disodium salt, β-Nicotinamide adenine dinucleotide phosphate sodium salt hydrate, EPPS and Dimethylsulfoxide (DMSO) were purchased from Sigma–Aldrich (St. Louis, MO, USA). The two enzyme preparations, Celluclast 1.5 L and Novozyme 188, were from Novozymes A/S (Bagsværd, Denmark). Celluclast 1.5 L derived from *Trichoderma reesei*, mainly harbouring CBH and EG activity [35], had an activity of 65 FPU/mL (FPU = filter paper unit) and 10 CBU/mL (CBU = cellobiose units). The FPU activity was determined by the NREL standardized filter paper assay. The CBU activity was determined by measuring glucose production on cellobiose at 50 °C, pH 4.8 [36]. Novozyme 188, harbouring BG from *Aspergillus niger*, had an activity of 856 CBU/mL. The hexokinase (420 U/mL) + glucose-6-phosphate dehydrogenase (G6P-DH) (210 U/mL) kit used for glucose analysis was purchased from Megazyme (Wicklow, Ireland). Fluorescein-5-EX succinimidyl ester was from Invitrogen (Carlsbad, CA, USA).

### 3.2. Immobilization of β-Glucosidase

Novozyme 188 was the source of the BG employed in this study. Prior to immobilization, the crude enzyme was centrifuged at 5000 g for 10 min and the precipitate was discarded. Protein concentration in the supernatant was determined by the Quick Start Bradford Protein Assay (Bio-Rad, Hercules, CA, USA) to be 88 mg/mL (with γ-globulin as reference). The enzyme was cross-linked using different concentrations of glutaraldehyde with/without bovine serum albumin (BSA) (Table 1), and then incubated with shaking at 100 rpm at 25 °C for 4 h to allow the crosslinking. For the crosslinking procedure, enzyme and glutaraldehyde were diluted by 200 mM EPPS, pH 10.5 (this buffer was used in order to make the final solution around neutral because Novozyme 188 is acidic). Next, a solution of 5% (w/w) sodium alginate in 5 mM sodium acetate, pH 4.8 with 0.02% sodium azide was prepared. The sodium alginate was then mixed with the cross-linked enzyme to yield a final alginate and enzyme concentration of 3.75% and 1.46 mg protein/mL, respectively. Following incubation at 4 °C overnight, the alginate-enzyme gel was dropped into a 200 mM CaCl_2_ solution in 5 mM, pH 4.8 sodium acetate buffer with stirring (the diameter of the aperture of the syringe tip was 3 mm). The beads were stirred for another 2 h at room temperature to improve toughness (curing) and then stored at 4 °C in 50 mM sodium acetate, pH 4.8 with 20 mM CaCl_2_ solution.

### 3.3. Analysis of Residual Activity

The residual β-d-glucosidase activity after glutaraldehyde treatment and after the curing and incubation steps was assayed according to a standard cellobiase assay procedure [36], see Section 3.1. In order to measure enzyme leakage, discarded solutions were collected and assayed after curing for 2 h and following subsequent buffer exchanges (repeated 3 times, using 50 mM sodium acetate buffer, pH 4.8 with 20 mM CaCl_2_ and incubated at 4 °C). The enzyme activities in the discarded buffer solutions were assayed immediately after collection. Residual enzyme activities in the calcium alginate were calculated as shown in Equation (1):

(Residual activity) = (Original activity) – (Lost activity due to cross-linking) – (Leakage)
(1)

### 3.4. Determination of K_m_ and V_max_ of BG

For free BG (FRBG), 7000 × diluted enzyme (0.122 CBU/mL) was reacted with 0.9, 2.25, 4.5, 9, 13.5, 18 and 22.5 mM of cellobiose and incubated at 50 °C with shaking at 650 rpm in a thermomixer for 8 min. For IMBG, the beads were mixed with different concentrations of cellobiose in a ratio of 1:9 (v/v, IMBG bead *vs.* substrate solution) and incubated at 50 °C with shaking at 100 rpm in water bath for 15 min. The reaction times of 8 and 15 min were selected to obtain a correct first order reaction rate. All reactions were stopped by heating at 100 °C for 5 min. The K_m_ and V_max_ values were derived from Hanes-Woolf plots.

### 3.5. Repeated Hydrolysis for Evaluating the Stability of the Recycled Enzyme

Using Avicel as the cellulose substrate (10%, w/v), hydrolysis reactions were carried out in 50 mM sodium acetate buffer solutions, pH 4.8 containing 0.02% sodium azide and 20 mM calcium chloride. The total volume was 25 mL. Dosage of Celluclast 1.5 L was 16 FPU/g-substrate while the dosage of BG was 13 CBU/g-substrate for FRBG or the same bead weight of IMBGs prepared under different conditions (*i.e.*, corresponding to the original 13 CBU/g-substrate). The negative control contained Celluclast 1.5 L only (*i.e.*, with no added BG). Reaction flasks were incubated in a 50 °C water bath with shaking. The individual batch reactions, each lasting 48 h with sampling for glucose yield after 9, 24, and 48 h, were repeated from scratch by recycling the beads with IMBG. To facilitate recycling and washing of the beads, the beads were maintained in a small cylinder cell, both sides were covered by nets with mesh size of 2 mm for substrate diffusion. Beads were washed by gentle water flushing, and then left for two rounds of 1 h in sodium acetate buffer pH 4.8 at 25 °C (buffer change between each round) to allow glucose and oligosaccharides to diffuse out of the beads. The washed beads were left in fresh buffer overnight at 4 °C before the next hydrolysis reaction was initated.

Samples withdrawn from reactions were deactivated via heating at 100 °C for 5 min. After cooling to room temperature, samples were centrifuged at 16,000 *g* for 2 min and the supernatant was subject to glucose analysis. Concentrations of liberated glucose were determined by the hexokinase + G6P-DH assay. Glucose yields (Y_glucose_) were calculated as detailed in Equation (2), where *W* indicates weight (hydrated glucose). *W_potential glucose_*: Weight of cellulose (*g*) × 180/(180 − 18):

(2) $$Y_{glu\text{cos}e}(\%)=\frac{W_{glu\text{cos}e}(g)}{W_{potential\;glu\text{cos}e}(g)}\times 100\%$$

All enzymatic hydrolysis reactions were run in two replications, and glucose analyses were done in duplicate. The data reported are given as averages ± s.d (calculated from the four data points for each).

### 3.6. Hydrothermal Pre-Treatment of Barley Straw

Barley straw was grown and harvested in 2006 on the island of Funen, Denmark. Thereafter, a sample was transported to Danish Oil and Natural Gas (DONG) Energy (Skærbæk, Denmark) for pre-treatment. The pre-treatment method consisted of a three-stage process, which involved heat treating the straw (initially at 16% by weight of dry matter (DM)) three times at progressively higher temperatures (60 °C, 15 min; liquids removed; 180 °C, 10 min; 195 °C, 3 min). After pre-treatment, the liquids were removed, and the dry matter obtained was 24% by weight [37]. Standard procedures for acid hydrolysis and compositional calculation analysis were carried out according to the standard procedure of the U.S. National Renewable Energy Laboratory [38].

### 3.7. Enzymatic Hydrolysis of Hydrothermally Pre-Treated Barley Straw

Pre-treated barley straw was cut to allow its passage through a sieve with an aperture of 2 mm (Endecotts, London, England). Enzymatic hydrolysis reactions on the pretreated barley straw were carried out in the same buffer as for Avicel hydrolysis, and reactions were run at pH 4.8, 50 °C [39]. The substrate concentration was 5% (w/v) and the enzyme dose was made up of 8 FPU/g-substrate Celluclast 1.5 L as well as 13 or 26 CBU/g-substrate BG. The diameter of the IMBG beads was around 4–5 mm. The dosage of IMBG was calculated based on that 63% activity remained after cross-linking (Figure 1); the IMBG dosage was 26 CBU/g-substrate. Flasks were incubated in a 50 °C water bath with shaking. Samples were removed from reactions at different time intervals during 72 h of hydrolysis for subsequent glucose analysis. Glucose yields were calculated according to Equation (2).

Enzymatic hydrolysis reactions were run in two replications, and glucose analyses were done in duplicate. The data reported are given as averages ± s.d (calculated from the four data points for each).

### 3.8. Visualization of Enzyme Distribution in Calcium Alginate by Confocal Laser Scanning Microscopy

Fluorescein-5-EX, succinimidyl ester was dissolved in DMSO to 10 mg/mL according to manufacturer’s instruction. Thin slices of IMBG were placed in 100 mM EPPS with 20 mM CaCl_2_, pH 8. Fluorescein was then added to the mixture, and the samples were incubated for 1.5 h at 25 °C. At the end of this interval, un-conjugated fluorescein was removed from the IMBG samples through repeated washings (5 times) using sodium acetate buffer. Microscopic observations and image acquisitions were performed on an LSM 510 confocal laser scanning microscope (CLSM) (Carl Zeiss, Jena, Germany) equipped with detectors and filter sets for monitoring fluorescence (500–550 nm). Images were obtained using a 63X/0.95W objective. Images were processed using Imaris software (Bitplane AG, Zürich, Switzerland).

## 4. Conclusions

Glutaraldehyde treated BG aggregates were successfully entrapped in 3.75% calcium alginate. Overall, in this way, 60% of BG residual activity could be recovered in the calcium alginate particles. The glutaraldehyde-cross linked, immobilized BG enzyme retained full activity during 20 times of re-use in extended enzymatic cellulose hydrolysis reactions of 48 h each. The performance of IMBG on lignocellulose hydrolysis was comparable to that of adding free enzyme at a corresponding dosage. BG aggregates in the matrix were visualized by CLSM. The CLSM images indicated that the glutaraldehyde cross-linking produced denser, immobilized BG aggregates than those without cross-linking and that the BG aggregates were evenly distributed in the calcium alginate matrix. In conclusion, the dosing of BG can be reduced significantly by this re-use, because of its high stability. However, application of BGs in the bioethanol industry, particularly for hydrolysis reactions at higher substrate concentrations, need to be investigated and improved in the future.

## Abbreviations

BG: (β-glucosidase)
IMBG: (immobilized β-glucosidase)
FRBG: (free β-glucosidase)
CLSM: (Confocal laser scanning microscopy)
GA: (glutaraldehyde)
BSA: (bovine serum albumin)
